# Expression and purification of epinecidin-1 variant (Ac-Var-1) by acid cleavage

**DOI:** 10.1007/s00253-024-13017-5

**Published:** 2024-01-26

**Authors:** Sivakumar Jeyarajan, Ansu Susan Peter, Aswathy Sathyan, Sukumar Ranjith, Indira Kandasamy, Senbagam Duraisamy, Prahalathan Chidambaram, Anbarasu Kumarasamy

**Affiliations:** 1 Microbial Biotechnology Laboratory, Department of Marine Biotechnology, Bharathidasan University, Tiruchirappalli, Tamil Nadu India; 2 Department of Pharmacology, University of Michigan, Ann Arbor, MI USA; 3 Department of Biotechnology, SRM University, Chennai, India; 4 Department of Biochemistry, Bharathidasan University, Tiruchirappalli, Tamil Nadu India

**Keywords:** Antimicrobial peptides, Epinecidin-1, Acid cleavage, Recombinant fusion protein, His tag

## Abstract

**Abstract:**

The demand for massive quantities of therapeutic active antimicrobial peptides (AMPs) is high due to their potential as alternatives to antibiotics. However, each antimicrobial peptide has unique properties, necessitating distinct synthesis and purification strategies for their large-scale production. In this study, we bio-synthesized and purified a functional enhanced variant of the AMP epinecidin-1, known as Ac-Var-1 (acid-cleavable variant-1). To generate the active peptide, we cloned the gene for Ac-Var-1 with acid-cleavable site (aspartic acid-proline) into the pET-32a expression vector, purified the fusion protein by His tag enrichment chromatography, and performed acid cleavage to release the active Ac-Var-1 peptide. After acid cleavage, the active Ac-Var-1 was purified and characterized by SDS-PAGE and mass spectrometry. The results from both techniques provided confirmation of the intactness of the purified Ac-Var-1. The Ac-Var-1 inhibited the growth of pathogenic *Escherichia coli* and *Staphylococcus aureus.*

**Key points:**

*• Epinecidin-1 is a well-known antimicrobial peptide having multipotential bioactivities.*

*• Epinecidin-1 variant is developed via the site-directed mutagenesis method to improve its structural stability and bioactivity.*

*• AC-Var-1 development is an economical and easy method to remove peptide from tag protein.*

## Introduction

Hospital-acquired infections caused by opportunistic bacterial pathogens, including those from the normal microbial flora, pose substantial challenges for immunocompromised patients. Current treatments often prove ineffective and costly, prompting research groups to seek novel antimicrobial compounds. The extensive use of antibiotics in public health and livestock management has contributed to the emergence of multi-drug resistant (MDR) superbugs, diminishing the efficacy of traditional antibiotics (Ventola 2015). These MDR pathogens have developed resistance to multiple antibiotics, leading to persistent and easily transmissible infections. Moreover, commonly used antibiotics frequently trigger adverse side effects like vomiting, diarrhea, gastrointestinal inflammation, and allergic reactions, some of which can be severe or even fatal. Additionally, the complex synthesis processes and high production costs associated with antibiotics present further hurdles. Consequently, numerous research groups are focusing on screening and developing novel antibiotic compounds that exhibit minimal side effects and can be produced through simpler procedures (Miethke et al. 2021; Jeyarajan et al. 2023a).

The rise in MDR has generated significant interest in exploring naturally occurring antimicrobial peptides (AMPs) as potential therapeutic alternatives. Antimicrobial peptides (AMPs) have demonstrated their effectiveness in combating invading pathogens (Drayton et al. 2021). These peptides are naturally occurring and can be found in various organisms across different kingdoms, playing a vital role in innate defense mechanisms. One notable advantage of AMPs is their reduced tendency to induce bacterial resistance. This is due to their unique mode of action, primarily targeting the physical barriers of bacteria. AMPs exhibit their effectiveness in neutralizing pathogens by directly interacting with the microbial membrane or disrupting crucial cellular processes. This unique mechanism hinders the development of resistance in pathogens, making AMPs an effective solution for combating microbial infections.

AMPs function by permeabilizing the cytoplasmic membrane, causing the leakage of ions and metabolites and dissipating the bacterium’s electrical potential (Anantharaman and Sahal 2010). This unique mechanism makes it challenging for bacteria to develop resistance against AMPs. These peptides are characterized by their short length, typically consisting of 10–50 amino acid residues. Their versatility as antibiotic alternatives stems from their ability to serve multiple functions. Firstly, they exhibit a broad spectrum of action, effectively targeting both Gram-positive and Gram-negative bacteria. Additionally, AMPs display a high level of selectivity towards bacterial membranes.

Over the past few decades, extensive research on antimicrobial peptides (AMPs) has significantly improved the options and prospects for combating the escalating threat of antibiotic-resistant microorganisms (George et al. 2022). Despite their notable advantages, such as a lower risk of resistance development and easy metabolization, the practical application of AMPs has been limited by several drawbacks, including high production costs, poor potency, instability, and inadequate selectivity (Jeyarajan et al. 2023a).

To address these limitations, one approach is to enhance the potency of natural AMPs by modifying their physico-chemical parameters. Numerous studies on AMPs, such as magainin (Zasloff 1987; Imura et al. 2008), alyteserin-2a (Conlon et al. 2012), cecropin (Shin et al. 2000), SMAP29 (Shin et al. 2001), LL-37 (Luan et al. 2014), and Gramicidin (Pavithrra and Rajasekaran 2020), have demonstrated the potential to remodel peptide sequences, imparting specific characteristics that improve stability and activity without hemolytic activity.

In our previous study (Jeyarajan et al. 2023a), we successfully addressed the limitations of native antimicrobial peptide (AMP) epinecidin-1 by designing improved variants with enhanced function and stability namely Var-1. To facilitate the overexpression and purification of this variant peptide, we employed a cloning strategy. Specifically, we cloned the gene encoding the Var-1 into the pET-32a vector. The pET-32a vector offered several advantageous features for the following reasons. It includes a fusion partner, thioredoxin, which aids in the translation of the recombinant protein. An enterokinase cleavage site is also present, allowing for the separation of the fusion protein and the active AMP peptide during downstream processing. Additionally, the vector contains a histidine tag that enables affinity purification of the recombinant peptide. Following the mentioned procedure, we successfully produced the active Var-1.

In this study following the same strategy, we added a formic acid-cleavage site (aspartate-proline (D-^↓^P)) between the carrier protein thioredoxin and the Var-1 sequence. We did this because formic acid is a weak acid and is required in very less amount to cleave the recombinant protein and release the active antimicrobial peptide. The advantage of formic acid cleavage over enterokinase treatment is there is no need for purified enterokinase enzyme for this procedure. Additionally, formic acid cleavage allows for direct treatment of the denatured fusion protein without the need to remove denaturants, simplifying the procedure and saving labor. Moreover, formic acid is readily available to buy. Since it is a weak acid, it can be neutralized after the reaction is complete and released to the environment without any hazard.

## Materials and methods

### Strains, vectors reagents, and enzymes

We used *Escherichia coli* (*E. coli*) strains DH5α (New England Biolabs (NEB), Ipswich, MA) and C43(DE3) (Sigma-Aldrich, St. Louis, MO) as host organisms for the cloning and overexpression of the fusion proteins. The plasmid vector pET-32a ( +) (Novagen-EMD Millipore, Burlington, MA) was used as the vector to clone the gene sequence of Ac-Var-1. Restriction enzymes and T_4_ DNA ligase were from NEB, Ipswich, MA. Plasmid extraction, gel extraction kits, and Ni–NTA resin were from Qiagen GmbH, Germany. Isopropyl β-D-thiogalactoside (IPTG), formic acid, and other chemical reagents were of analytical grade purchased from Sigma-Aldrich, St. Louis, MO.

### Design of Ac-Var-1 and structural parameter analysis

Var-1 peptide contains an amino acid sequence (FIFKIIKGLFKAGKMIKGLV). Acid-cleavable site aspartate-^↓^proline (D^↓^P) was added to the N-terminus of Var-1 so that the sequence will be (D^↓^P FIFKIIKGLFKAGKMIKGLV) and termed as acid-cleavable Var-1 (Ac-Var-1). The downward-pointed arrow indicates the acid-cleavable position. The secondary structural features were calculated using i-TASSER (http://zhanglab.ccmb.med.umich.edu/I-TASSER/). The physico-chemical properties such as hydrophobicity (GRAVY), therapeutic index (Ti), Boman index (kcal/mol), and Wimley-White partition scale were calculated from the APD database tool (http://aps.unmc.edu/AP/prediction/actionInput.php) and Mutator tool site (http://split4.pmfst.hr/mutator/).

### Synthesis and cloning of DNA fragment encoding Ac-Var-1 into pET-32a vector with the acid-cleavage site

In this study, we have used an assembly PCR method as given by Jeyarajan et al. (2023a) for the synthesis of the Ac-Var-1 gene. The coding fragment of the peptides was assembled in sequential steps of PCR with two sets of primers as given in Table 1. The PCR-amplified Ac-Var-1 gene was cloned into the pET-32a ( +) vector between *Eco*RI and *Xho*I restriction sites. The obtained clone was named pET-32a-Ac-Var-1. The presence of insert was confirmed by restriction digestion analysis. By cloning into the pET-32a vector, the peptides can be expressed as thioredoxin fusion protein in *E. coli*.

**Table 1:** The oligomers used for generation of epinecidin-1 and Ac-Var-1 gene by overlapping extension PCR

	Oligomer sequence (5′-3′)
Ac-Var-1	
Set 1	CATATCATTAAAGGCCTGTTTCATGCGGGCAAAATGAT
	CCTGGTCACCAGGCCATGGATCATTTTGCCCGCATGAAAC
Set 2	CGCGT*GAATTCGATCCGTTTATCTTCAAGATCATTAAAGG
	TATA^#^*CTCGAG*TTAATGTCGTCTCCTGGTCACCAG

The overlapping complementary sequences are underlined. *EcoRI and ^#^*Xho*I restriction enzyme recognition sites are bold and italicized.

### DNA sequencing

All the positive constructs containing the Ac-Var-1 gene of the insert were sequenced using an Applied Biosystems Automated 3700 DNA Analyzer with Big Dye Terminator chemistry. The sequence chromatogram was analyzed with Finch TV software (Geospiza Inc- Seattle-WA).

### Optimization of time points for overexpression of recombinant Trx-Ac-Var-1

The recombinant protein expressed from pET-32a-Ac-Var-1 was named Trx-Ac-Var-1. For the time point optimization studies, the clone was transformed into *E. coli* C43(DE3) protein expression host strain. The transformed cells were plated on Luria Bertani (LB) agar plates containing ampicillin (50 µg/ml). The single colony was picked from the plate and inoculated in flasks containing 20 ml LB broth to analyze the overexpression profile. The flask was incubated at 30 °C in an orbital shaker at 250 rpm, cultured till mid-log phase (optical density OD_600_ of 0.6–0.8) and aliquots of 1 ml were removed as uninduced control. The O.D of un-induced control was measured for protein normalization. The remaining culture was induced with 0.3 mM IPTG. This aliquoted uninduced control sample was spun at 10,000 × g for 10 min at 4 °C in a centrifuge to pellet the cells. The supernatant was removed by aspiration and the pellet was stored at − 20 °C. For induced controls, aliquots of 1 ml culture were taken out at 2nd, 3rd, and 4th h, respectively, their OD was measured and pelleted by centrifugation at 10,000 × g for 10 min at 4 °C, and the supernatant was aspirated off. The pellets were stored at − 20 °C until the samples were ready for lysis. The uninduced control and induced samples were removed from the − 20 °C freezer. An amount of Buffer (10 mM Tris–HCl (pH 8.0), 100 mM NaH_2_PO_4_, and 1% SDS) determined by factor (270/(10 × value of OD_600_)) as described in Novagen pET system manual was added to the pellet to normalize the sample protein concentrations. The mix was vortexed until the entire cell pellet was in solution. All samples were centrifuged at 14,000 × g for 10 min at 4 °C to pellet cell debris, and the supernatant was transferred to a fresh tube for loading in a 15% SDS-PAGE.

### Preparation of cell lysate and protein solubility analysis

The recombinant pET-32a-Ac-Var-1 clone in *E. coli* C43 (DE3) cell was induced and harvested by centrifugation at 4000 × g and stored overnight at − 20 °C. The cell pellets were suspended in lysis buffer (50 mM NaH_2_PO_4_, pH 8.0, 300 mM NaCl) with lysozyme to a final concentration of 1 mg/ml and incubated on ice for 30 min, followed by sonication for 5 times with a burst duration of 15 s each. The sonicated lysates were centrifuged at 14,000 × g for 10 min, the supernatants containing the soluble proteins were collected into fresh tubes, and the pellet containing the insoluble proteins was solubilized by 0.1% SDS and loaded in 15% SDS-PAGE for evaluating the solubility of the overexpressed recombinant proteins.

### Purification strategies of the Trx-Ac-Var-1 fusion protein for formic acid cleavage

The protein overexpressed from pET-32a-Ac-Var-1 is named Trx-Ac-Var-1 since it is a fusion protein with thioredoxin. Trx-Ac-Var-1 got accumulated in inclusion bodies. The inclusion body was lysed with lysis buffer containing 8 M Urea and 50 mM Tris–HCl (pH 8.0) in the presence of protease inhibitor 1 mM PMSF by sonication. The lysate was centrifuged at 14,000 × g for 10 min at 4 °C to pellet the cell debris. The supernatant was made to bind with 5 ml of Ni–NTA slurry pre-equilibrated with lysis buffer. The overexpressed fusion protein bound to the column with a 6 × histidine tag present between the N- and C-terminal domains. The Ni–NTA bound protein column was washed with lysis buffer initially and further with two-bed volumes of wash buffer (lysis buffer containing 50 mM imidazole). The enriched bound protein was eluted with the two-bed volume of elution buffer (lysis buffer containing 250 mM imidazole at pH 8.0). The fractions collected from the column were loaded on 15% Tris–Glycine SDS-PAGE.

The imidazole eluted Trx-Ac-Var-1 was directly treated with 50% formic acid (v/v) at 50 °C for 24 h (Li et al. 2006, 2007; Ramos et al. 2013). After cleavage, formic acid was removed in a Vacufuge (Eppendorf speed vac 5301, GmBH, Germany) till the acid gets evaporated. The lyophilized powder was buffer exchanged with Phosphate Buffered Saline pH 7.4 (PBS) using PD-10 Cytiva 17–0851-01 column (Sigma-Aldrich, St. Louis, MO) according to the manufacturer’s protocol. The acid-cleaved thioredoxin carrier and active Ac-Var-1 peptide mix were collected in a final volume of 2.5 ml. This mix was made to bind Ni–NTA slurry pre-equilibrated with the same solution for 1 h at 4 °C. This was done to make the histidine-tagged thioredoxin to bind to the Ni–NTA column and get the active Ac-Var-1 peptide in the unbound fraction. The fractions harvested from the Ni–NTA column were analyzed on 15% tris-tricine SDS-PAGE. The gel was processed for silver staining with the protocol described by Bio-Rad Life Sciences laboratories, Hercules, CA. Briefly, the tris-tricine gel after completion of the electrophoretic run was immersed in a fixing solution (40% methanol (v/v) and 10% acetic acid (v/v)) for 4 h. After fixation, the gel was washed with 10% ethanol (v/v) for 30 min. The ethanol wash was followed by treating with oxidizer solution (1% potassium dichromate (w/v) and 0.5% nitric acid (v/v)) for 10 min. Consequent to oxidizer treatment, the gel was continuously washed with de-ionized water until the yellow coloration was removed. Subsequently, the gel was dipped in 0.2% silver nitrate (w/v) for 30 min. The silver nitrate-treated gel developer solution (2.5% sodium carbonate (w/v) and 0.02% (v/v) formaldehyde) was added till the bands appeared. After the appearance of bands, the development was stopped by incubation with 5% acetic acid (v/v) for 5 min and then transferred to de-ionized water and photographed.

### Purity and molecular mass determination

The molecular mass and homogeneity of recombinant Trx-Ac-Var-1 and the active Ac-Var-1 peptide were determined using matrix-assisted laser desorption/ionization time-of-flight mass spectrometry (MALDI-TOF MS). For the Trx-Ac-Var-1, a linear time-of-flight mode was performed and for the acid-digested Ac-Var-1 peptide both linear and reflector mode was performed for high resolution using Applied Biosystems/MDS SCIEX 4800 MALDI TOF/TOF™ Analyzer (Waltham, MA, USA). The samples were run in positive detection mode using alpha-cyano-4-hydroxycinnamic acid as the matrix. Internal mass calibration of the instrument with known standards was used.

### Antimicrobial activity of fusion and acid-cleaved Ac-Var-1

The antimicrobial potential of the AMPs was determined using the agar well diffusion method (Bauer et al. 1966) with slight modifications. Mueller–Hinton agar (MHA) was dissolved in distilled water to 38 g/l. The sterilized MHA was poured into sterile petri dishes and allowed to solidify. Using sterile cotton swabs, the pathogens *Escherichia coli* (*E. coli*) (MTCC 2939) and *Staphylococcus aureus* (*S. aureus*) (ATCC 25923) were swabbed onto MHA and allowed to dry; a 7 mm diameter hole was punched aseptically on the swabbed plate using a sterile cork borer. Fifty microliter at 100 µg concentration was pipetted into the pierced hole. The buffer in which the proteins are present (50 mM Tris–HCl (pH 8.0)) served as negative control and ampicillin (100 µg/ml) served as positive control. Recombinant purified thioredoxin was used as an additional control. Buffer-exchanged recombinant Trx-Ac-Var-1 and acid-cleaved Trx-Ac-Var-1 were pipetted into the pierced hole individually. In-house purified recombinant epi-1 and var-1 as mentioned in (Jeyarajan et al. 2023a) were also used as controls for peptide activity. The plates were then placed in an upright posture at 37 °C in an incubator overnight to evaluate the activity of AMPs. The diameter of the inhibition zone was measured in millimeter (mm) and plotted as mean ± standard deviation. The experiments were performed in triplicates. Tukey’s multiple comparisons test (two-way ANOVA) was performed for statistical analysis and significance representations are ns, non-significant; *p < 0.5, **p < 0.1, ***p < 0.01, and ****p < 0.001.

## Results

### In silico characterization of Ac-Var-1

The primary amino acid sequence of Var-1, FIFKIIKGLFKAGKMIKGLV, as reported in the study by Jeyarajan et al. (Jeyarajan et al. 2023a) was derived from the epinecidin-1 parent peptide. Among the variants described in that study, Var-1 was chosen for large-scale production with an acid-cleavable strategy due to its enhanced stability and antimicrobial potential. An acid-cleavable site was added to the N-terminus of the Var-1 peptide named Ac-Var-1 (acid-cleavable Var-1) and has the sequence D^↓^P FIFKIIKGLFKAGKMIKGLV.

**Fig. 1:**
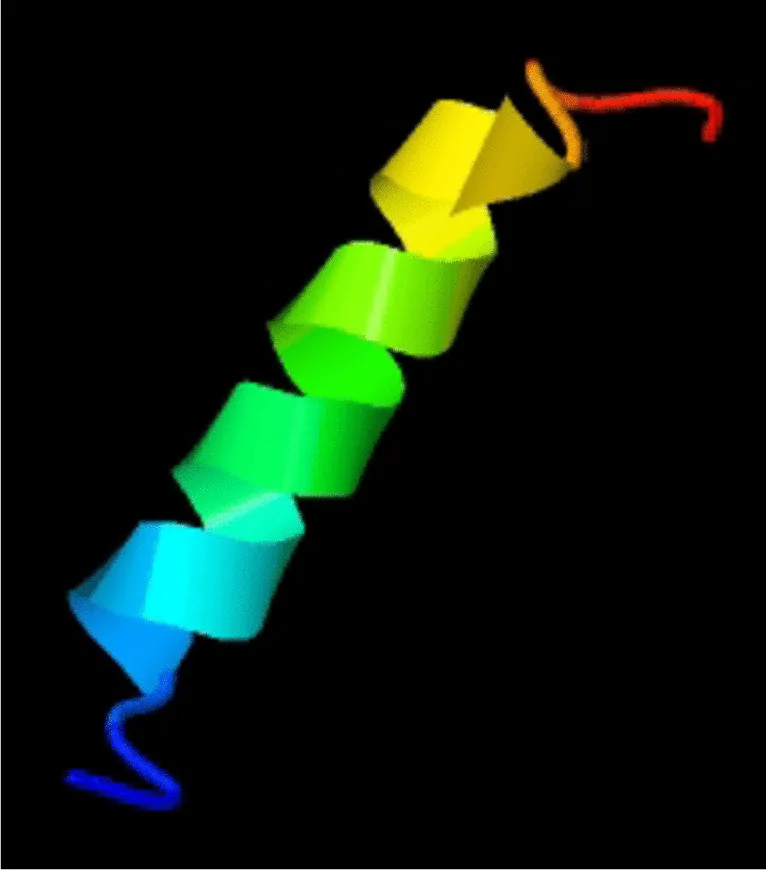
Secondary structure model generated by i-TASSER with TM score 0.64 ± 0.13

The i-TASSER program was used to obtain secondary structure data of the peptide, demonstrating that the peptide forms an alpha helix (71.4%) and a residual coiled structure (Table 2). The (Template Modeling) score further confirmed the amphipathic helical structure, aligning with its antimicrobial function (Dathe and Wieprecht 1999; Thankappan et al. 2013; Kabelka and Vácha 2021) (Fig. 1). Physico-chemical properties obtained from in silico analysis also showed the peptide’s amphipathic nature (Table 3). The peptide has a grand average of hydropathy (GRAVY) value of 0.933. The Boman index, which signifies the peptide’s potential to bind with lipids or proteins, demonstrated that Ac-Var-1 possesses the capacity to connect with the microbial membrane.

**Table 2:** Secondary structural characteristics calculated from i-TASSER

S. No		Sequence (Ac-Var-1)																					Length	Net charge	α-Helix (%)	No. and (%) of lysine	No. of non-polar residues	Theoretical pI (isoelectric point)
1	Position	1	2	3	4	5	6	7	8	9	10	11	12	13	14	15	16	17	18	19	20	21	21	+ 8	71.4	5 (23.8)	11	10.61
2	Primary sequence	P	F	I	F	K	I	I	K	G	L	F	K	A	G	K	M	I	K	G	L	V						
3	^$^Predicted Secondary structure	C	H	H	H	H	H	H	H	H	H	H	H	C	C	H	H	H	H	C	C	C						
4	Confidence score for secondary structure	9	1	6	9	9	9	9	9	9	9	8	7	1	1	4	6	6	1	1	5	9						
5	*Predicted solvent accessibility	7	3	3	2	4	1	1	4	2	2	1	4	3	2	4	3	2	5	4	3	7						

^$^C, Coil; H, alpha Helix

*Values range from 0 (buried residue) to 9 (highly exposed residue)

**Table 3:** Physico-chemical characteristics calculated from the primary amino acid sequence of Ac-Var-1

Sequence (Ac-Var-1)	TM score	GRAVY	Boman index (kcal/mol)	Wimley-White hydrophobicity	Therapeutic index (Ti)
PFIFKIIKGLFKAGKMIKGLV	0.64 ± 0.13	0.933	− 1.03	− 0.31	6.01

The Wimley-White hydrophobicity value, which measures the energetics required for peptide transfer across membrane, was calculated as − 0.31 for Ac-Var-1. This value closely resembles those of peptides like melittin, magainin 2, and mastoparan X, which all feature an amphipathic alpha helix (Almeida 2014). The therapeutic index (Ti) is an estimate derived from experimental measurements of hemolysis from a library of peptides used as training sets, evaluated in freshly isolated human erythrocytes from healthy donors. Typically, a Ti of 85 corresponds to 50% hemolysis (Kamech et al. 2012). The Ti value for Ac-Var-1 is 6.01, indicating that Ac-Var-1 is not hemolytic (Juretić et al. 2009). There is also experimental proof from the previous report (Jeyarajan et al. 2023a).

### Cloning of fusion Ac-Var-1 gene with thioredoxin in pET-32a vector

**Fig. 2:**
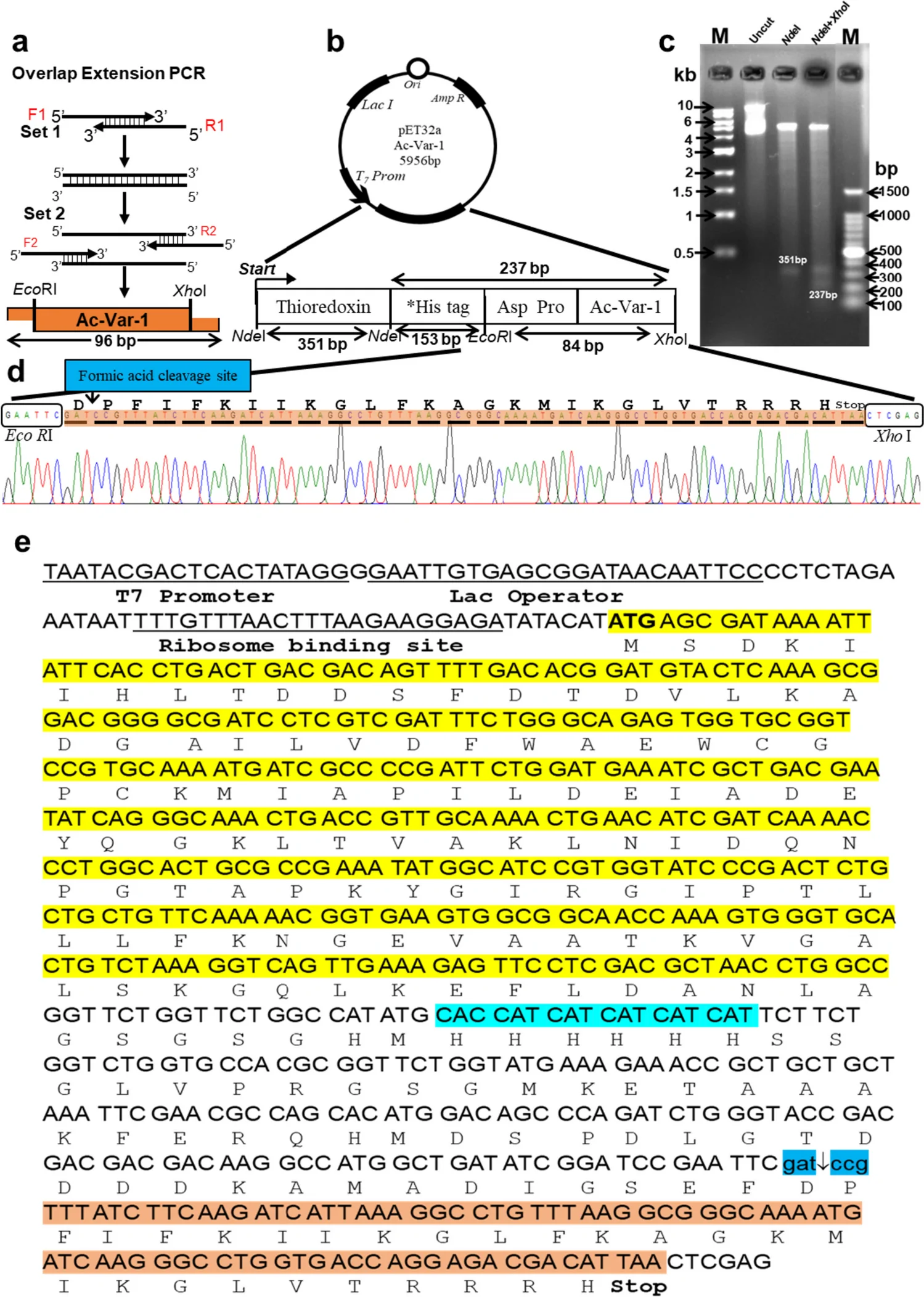
Construction of the Ac-Var-1 expression vector. (**a**) The Ac-Var-1 gene was synthesized using two rounds of overlapping PCR. An amplicon of 96 bp containing the formic acid-cleavage site and Ac-Var-1 gene flanked by *Eco*RI and *Xho*I restriction enzyme sites is shown. Then, the resulting gene fragment was cloned into the pET-32a vector for subsequent overexpression. (**b**) Subsequent to digestion with *Eco*RI and *Xho*I restriction enzymes, the gene was ligated into the pET-32a vector. This ligation resulted in an intact gene of 84 bp, which is in-frame with the thioredoxin gene of the pET-32a cassette vector and has an acid-cleavage site preceding the start of the Ac-Var-1 gene. The resulting pET-32a-Ac-Var-1 clone had a size of 5956 bp. The vector map indicates the sizes of the fragments within the restriction sites. Please note that the *His Tag represents the 6 × histidine tag alone, while the additional nucleotides from the vector are not shown. (**c**) The agarose gel image illustrates the results of the restriction enzyme analysis performed on pET-32a-Ac-Var-1 using *Nde*I and *Xho*I. The gel comprises lanes for the uncut plasmid and a molecular weight marker for reference. The last two lanes exhibit the restriction digested pET-32a-Ac-Var-1 samples, one treated with *Nde*I alone and the other with a combination of *Nde*I and *Xho*I. In the *Nde*I lane, a distinct band at 351 bp corresponds to the released thioredoxin gene fragment. Meanwhile, the *Nde*I + *Xho*I lane exhibits an additional fragment at 237 bp, which includes the His-tag, acid-cleavage site, and the Ac-Var-1 gene along with the thioredoxin gene fragment derived from the vector backbone. (**d**) The electrophoretogram excerpt displays the sequencing results of pET-32a-Ac-Var-1 performed using the T_7_ promoter primer. The image only includes the electrophoretogram region that spans between the *Eco*RI and *Xho*I restriction sites. Above the electrophoretogram, the triplet codons encoding Ac-Var-1 are indicated and underlined. The letters correspond to the open reading frame of the translated product of Ac-Var-1. The amino acid sequence encoding the acid-cleavage site, specifically aspartate and proline, is marked by a downward-pointing arrow adjacent to the *Eco*RI site. (**e**) The schematic representation depicts the nucleotides and the translated Open Reading Frame (ORF) of the pET-32a-Ac-Var-1 and Trx-Ac-Var-1 fusion AMP. Upstream of the thioredoxin gene, the 5′-untranslated nucleotides are shown, including the T_7_ promoter sequence, lac operator, and ribosome binding site. The thioredoxin protein, originating from its methionine start codon, is illustrated in yellow. Downstream of the thioredoxin, the His tag is represented in light blue, followed by the acid-cleavage site with the Asp-Pro sequence represented in dark blue. Finally, the Ac-Var-1, located after the acid-cleavage site, is depicted in orange color

The Ac-Var-1 gene was synthesized using overlap extension PCR and then cloned into the pET-32a vector, specifically within the *Eco*RI and *Xho*I restriction sites. This resulting construct was named pET-32a-Ac-Var-1. The pET-32a-Ac-Var-1 construct, illustrated in Fig. 2b, contains the carrier protein thioredoxin, a 6xHis-tag for affinity purification, and a formic acid-cleavage site for the release of the active peptide. To confirm the authenticity of the sequence and the orientation of the insert, restriction digestion analysis and DNA sequencing were performed. As shown in Fig. 2c, the pET-32a-Ac-Var-1 construct was digested separately with *Nde*I and with both *Nde*I and *Xho*I. When digested with *Nde*I alone, a 351 bp fragment corresponding to the thioredoxin carrier protein gene was released. Digestion with both *Nde*I and *Xho*I resulted in the release of the thioredoxin carrier protein, along with downstream genes such as the His-tag, acid-cleavable site (D^↓^P), and Ac-Var-1, with a total length of 237 bp. Therefore, the restriction digestion using *Nde*I and *Xho*I confirmed both the length of the sequence and the orientation of the insert. This was further confirmed by DNA sequencing. The sequencing results are depicted in Fig. 2d, which confirm that the gene was correctly inserted into the pET-32a vector between the *Eco*RI and *Xho*I sites. To obtain the protein sequence, the experimentally determined DNA sequence of the clone was analyzed using the EXPASY translate tool. The resulting protein translation perfectly matched thioredoxin and the Ac-Var-1 gene. The fusion protein sequence was named Trx-Ac-Var-1 and predicted to have a molecular weight of 21,150.2 Da. The fusion sequence translated from the pET-32a-Ac-Var-1 construct is represented schematically in Fig. 2e. After validating the clone, it was transformed into *E. coli* C43(DE3) expression host.

### Overexpression analysis and His tag enrichment of Trx-Ac-Var-1

**Fig. 3:**
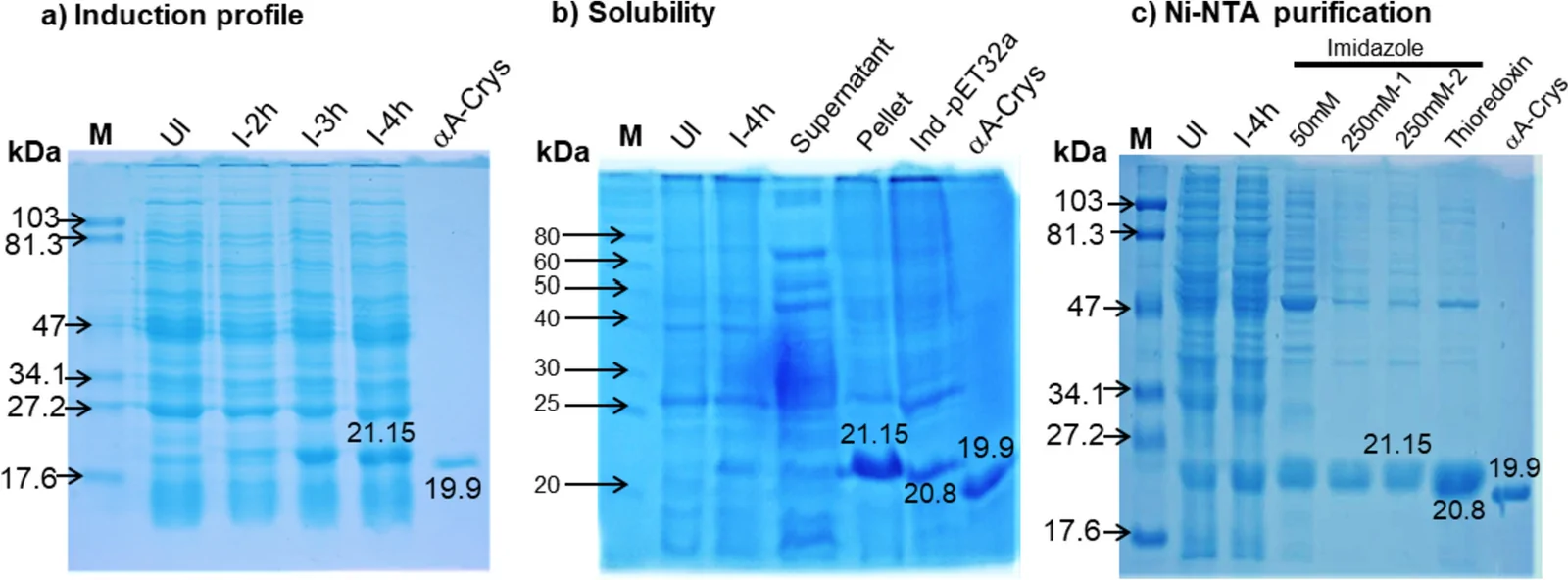
SDS-PAGE profiles for induction, solubility, and Ni–NTA purification of Trx-Ac-Var-1. **a** Time-course analysis of Trx-Ac-Var-1 overexpression in *E. coli* C43(DE3) whole-cell lysates. M represents the protein marker (Bio-Rad Cat# 161–0305). UI represents the uninduced lysate. I-2 h, I-3 h, and I-4 h represent lysates collected from cells induced with 0.3 mM IPTG at 2, 3, and 4 h post-induction, respectively. The αA-Crys lane shows in-house purified recombinant αA-crystallin protein of 19.9 kDa used as an additional molecular weight control. Overexpression of clones is indicated by a gradual increase in band intensity in the induced lanes, slightly above 19.9 kDa, but not in the uninduced lanes. **b** Assessment of the solubility of overexpressed Trx-Ac-Var-1. Lane M represents the protein marker (NEB, USA). Whole-cell lysates of the uninduced (UI) and induced (I-4 h) fractions are shown. The I-4 h lane exhibits a distinct band slightly above 19.9 kDa, which is not observed in the UI lane. The supernatant lane displays the soluble fraction obtained after lysis, sonication, and centrifugation. The pellet lane represents the insoluble cell debris dissolved with 0.1% SDS. The Ind pET-32a lane represents the lysate of 0.3 mM IPTG-induced pET-32a empty vector used as a control to distinguish between thioredoxin and thioredoxin-Ac-Var-1 after 4 h of induction. The αA Crys lane shows in-house produced recombinant αA crystallin of 19.9 kDa as an internal control. This figure confirms the presence of a small amount of protein in the soluble form and a large amount of protein in the inclusion bodies. Since a greater amount of protein is present in the inclusion bodies, they were used for further downstream processing. **c** Ni–NTA purification of Trx-Ac-Var-1. M represents the molecular weight marker (Bio-Rad). Lanes UI and I-4 h show equal portions of the cell lysate before and after IPTG induction. The 50 mM lane represents the imidazole wash fraction. The subsequent two lanes represent the 250 mM imidazole elution fractions. Purified thioredoxin and αA crystallin are shown in the last two lanes

Initially, time point optimization was performed for Trx-Ac-Var-1 as shown in Fig. 3a. Trx Ac-Var-1 clone in *E. coli* CD43DE3 was grown to mid-log phase and induced with 0.3 mM IPTG. An equal number of cells were collected before induction and 2, 3, and 4 h post induction. The harvested cells were centrifuged and lysed with SDS for PAGE analysis. The expected theoretical molecular weight of Trx-Ac-Var-1 was calculated to be 21.15 kDa, so a strong band between 17.6 and 27.2 kDa was anticipated. The time-course analysis of protein expression, specifically induced with 0.3 mM IPTG, revealed the appearance of a novel band exhibiting increasing intensity over time in the range of 17.6 to 27.2 kDa. This observation suggests that the expression system is producing an additional protein alongside the bacterial proteins. This gel image confirmed the overexpression of the fusion protein, and the optimum time was established as 4 h. An additional control, in-house purified alpha A crystallin with a known molecular weight of 19.9 kDa was added.

To determine the accumulation localization of Trx-Ac-Var-1 after 4 h of induction with 0.3 mM IPTG, solubility analysis of the fusion protein was performed, as shown in Fig. 3b. The harvested bacterial cells were initially lysed using a buffer containing lysozyme. After the lysozyme lysis, the lysate was centrifuged to separate the soluble protein fraction (supernatant) and insoluble inclusion bodies (pellet). Then, the insoluble pellet was solubilized using 0.1% SDS. The soluble and insoluble fractions were mixed with gel loading buffer and analyzed using SDS-PAGE. An intense 21.15 kDa band was observed in the lane labeled “pellet,” indicating that the fusion Trx-Ac-Var-1 accumulated in the insoluble inclusion bodies.

Once it was established that Trx-Ac-Var-1 was found in the insoluble fraction, the inclusion bodies were treated with denaturing lysis buffer to dissolve the protein. The resulting lysate was then applied to a Ni–NTA column that had been pre-equilibrated with the same lysis buffer. This allowed Trx-Ac-Var-1 to bind to the nickel ions on the column via its histidine tag. To eliminate non-specifically bound proteins, a washing step was conducted using wash buffer (lysis buffer containing 50 mM imidazole). The wash fraction contained a larger amount of other proteins compared to Trx-Ac-Var-1. After washing, the strongly bound Trx-Ac-Var-1 was eluted using elution buffer (lysis buffer containing 250 mM imidazole). Samples of the collected fractions were loaded onto SDS-PAGE gels, as depicted in Fig. 3c. To verify the molecular weight and purity of Trx-Ac-Var-1, standard molecular weight markers, uninduced and induced lysates, as well as purified thioredoxin and alpha-crystallin with known molecular weights, were included as controls. A yield of approximately 6.5 mg/l of the culture medium was obtained.

### Ac-Var-1 peptide release and purification

**Fig. 4:**
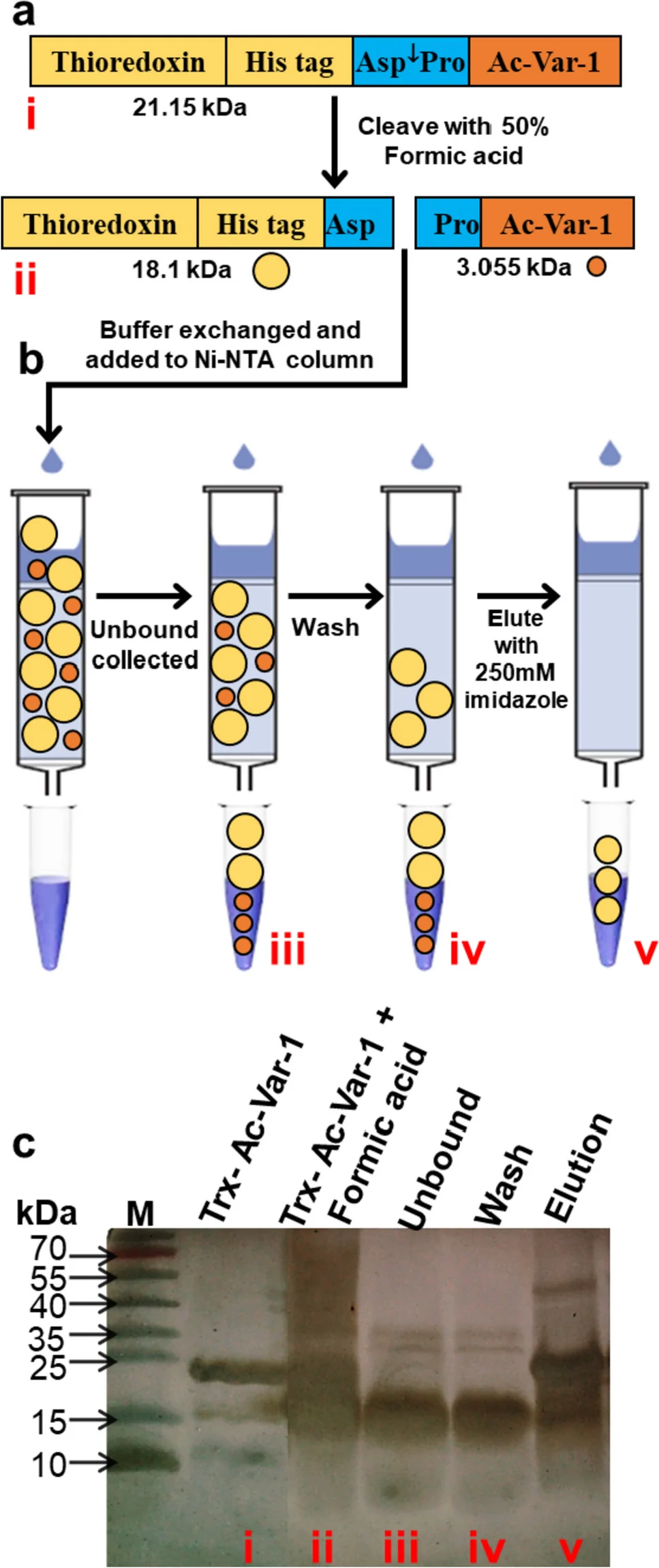
Expression strategy of Trx-Ac-Var-1 and subsequent acid cleavage. (**a**) The arrangement of the regions of the construct is shown (i), including the acid-cleavage site. The acid-cleavage site is located between the thioredoxin-His tag and the Ac-Var-1. The peptide Ac-Var-1, released after treatment with 50% formic acid, is depicted in (ii). The cleavage results in thioredoxin and His tag remaining as one part (represented by a yellow circle), while the active Ac-Var-1 peptide as the other part (represented by a small orange circle). As the his-tag is present in the thioredoxin part, it will bind to the Ni–NTA column, and the active Ac-Var-1 will be present in the unbound fraction. The expected sizes of the resulting fragments are also indicated, with 18.1 kDa for the entirety of thioredoxin and His tag and 3.055 kDa for the Ac-Var-1 peptide. (**b**) The schematic illustrates the steps involved in purifying the Ac-Var-1 from thioredoxin. Roman numerals denote the proteins and peptide fragments that were analyzed using SDS-PAGE. (**c**) The fractions loaded onto the SDS-PAGE were stained with silver are shown. (i) Whole-length Trx-Ac-Var-1, (ii) formic acid-cleaved mixture buffer exchanged with PBS, (iii) unbound fraction, (iv) wash fraction, and (v) thioredoxin eluted from the Ni–NTA column

The His tag enriched Ac-Var-1 containing fusion protein, without further concentration or buffer exchange, was cleaved in 50% formic acid (v/v) (e.g., 19.5 ml 88% formic acid and 15 ml fusion protein) at 50 °C for 24 h. Figure 4a(i) shows the schematic of steps involved in the purification of the Ac-Var-1. After cleavage, the Pro residue was left at the N-terminus of Ac-Var-1 (Fig. 4aii). Consequent to treatment with formic acid, the cleaved products were lyophilized and buffer exchanged with PBS. The His-tagged thioredoxin carrier and residual undigested fusion proteins were made to bind the Ni–NTA resin (Fig. 4b) and the active Ac-Var-1 was collected in the unbound fraction. These fractions were loaded on Tricine-SDS PAGE and stained with silver (Fig. 4c). The active Ac-Var-1 was seen in the unbound fraction according to our hypothesis; however, the carrier thioredoxin protein is also seen in the unbound fraction as a contaminant. The last lane in Fig. 4c is the elution of the carrier protein thioredoxin from the Ni–NTA column. It shows the presence of un-digested fusion protein and digested carrier protein thioredoxin. The treatment of Trx-Ac-Var-1 (6.5 mg) with formic acid and subsequent desalting yielded 4 mg of a mixture consisting of digested thioredoxin and active peptide. Upon passing this mixture through a Ni–NTA column, the unbound fraction finally resulted in 0.8 mg of the active peptide Ac-Var-1.

### Characterization of Trx-AC-Var-1 and Ac-Var-1 by mass spectrometry

**Fig. 5:**
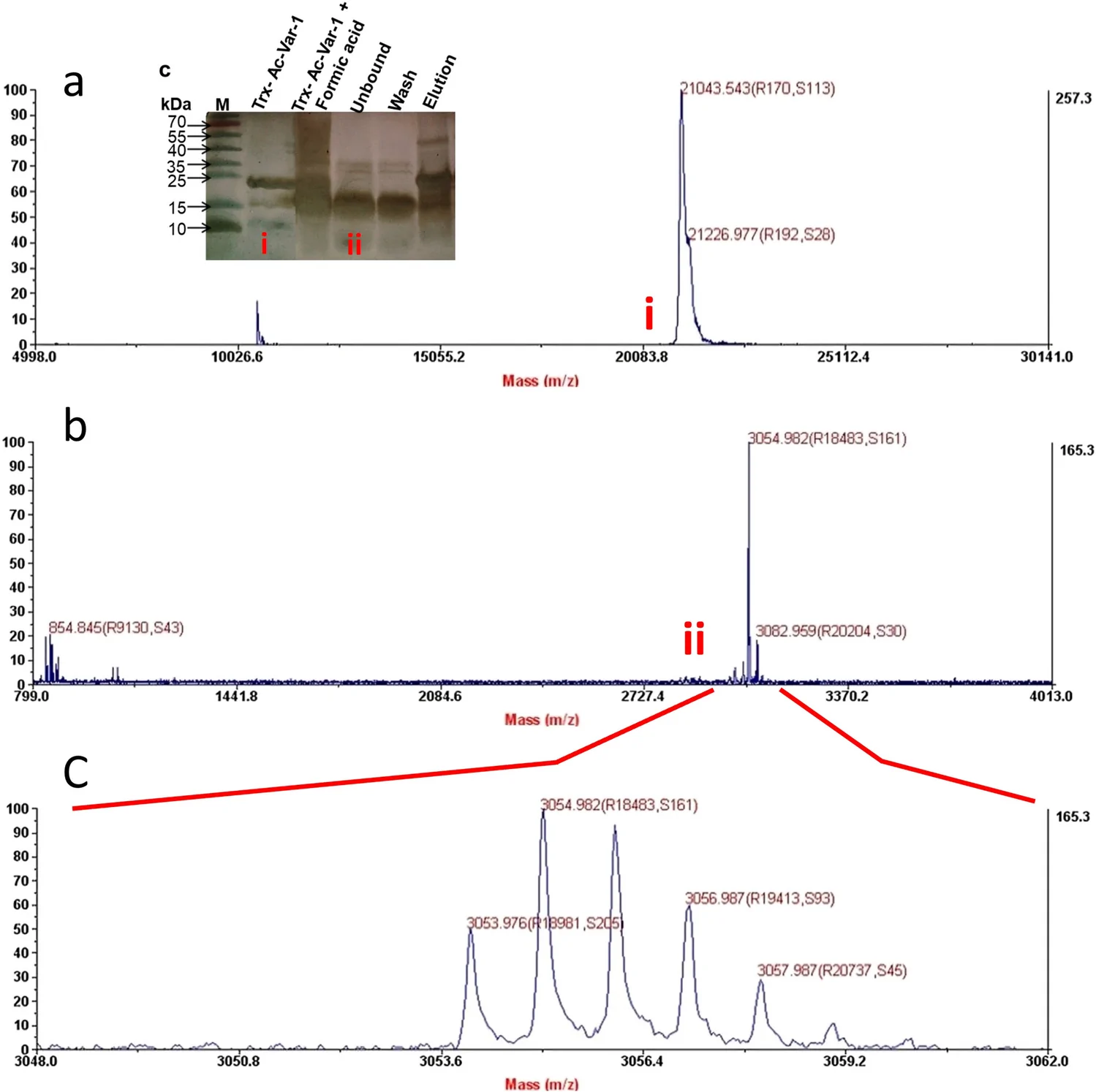
Mass spectrometry results of the full-length fusion Trx-Ac-Var-1 and acid-cleaved Ac-Var-1. After Ni–NTA purification, both the fusion Trx-Ac-Var-1 and the acid-cleaved Ac-Var-1 were subjected to mass spectrometry to characterize their integrity and molecular weight. (**a**) The mass spectra of Trx-Ac-Var-1. Theoretical is MW 21150.21 Da and experimental molecular weight is 21,043.54 Da. The inset shows the SDS-PAGE with the characteristic electrophoretic pattern. The compared mass spectrum and electrophoretic mobility are indicated by Roman numerals. (i) for Trx-Ac-Var-1 and (ii) for Ac-Var-1. (**b**) The mass spectrum of the acid-cleaved Ac-Var-1 is shown. The spectrum was focused on the range of 799 to 4013 Da to obtain the mass of the small peptide Ac-Var-1 in linear mode. The same peptide was characterized by SDS-PAGE, and its corresponding mass is indicated in the spectrum (ii). (**c**) The active peptide Ac-Var-1 was characterized using reflector mode for high resolution

MALDI-TOF analysis was performed for authentication of the exact molecular weight of the fusion protein and active peptide. Their mass is given in Table 4 and the spectrum is given in Fig. 5. Silver-stained gel is shown inset for reference. The fractions identified in mass spectrometry are shown in Roman numerals pointing to the electrophoretic pattern and mass spectrum. Trx-Ac-Var-1 and Ac-Var-1 are shown in Roman numerals i and ii. The experimentally determined molecular weight of Trx-Ac-Var-1 whole protein was found to be 21,043.54 and the theoretical molecular weight was calculated to be 21,150.21. The difference in molecular weight of the predicted and experimental molecular weight is 106.67 Da (one amino acid difference). This difference is within the allowed range (Brenton and Godfrey 2010); hence, a good correspondence with theoretical predicted molecular weight was observed. For the Ac-Var-1, a perfect match for the theoretical (3055.0 Da) and experimental molecular weight (3054.98 Da) is seen with a very minimal difference of 0.8 Da. The linear mode (Fig. 5b) shows a very sharp intense peak and the reflector mode (Fig. 5c) done for high resolution shows accurate and precise peaks in the range 3053 to 3057 Da which is a very credible co-relation to the calculated and experimental molecular weight.

**Table 4:** Theoretical molecular weight and experimentally determined molecular weight of Trx-Ac-Var-1 and acid-cleaved Ac-Var-1

	Amino acid sequence	Theoretical molecular weight (Da)	Experimental molecular weight (Da)
Trx-Ac-Var-1	MSDKIIHLTDDSFDTDVLKADGAILVDFWAEWCG PCKMIAPILDEIADEYQGKLTVAKLNIDQNPGTAPK YGIRGIPTLLLFKNGEVAATKVGALSKGQLKEFLD ANLAGSGSGHMHHHHHHSSGLVPRGSGMKETAA AKFERQHMDSPDLGTDDDDKAMADIGSEFD PFIFKIIKGLFKAGKMIKGLVTRRRH	21,150.21	21,043.54
Ac-Var-1	PFIFKIIKGLFKKGKMIKGLVTRRRH	3055.8	3054.98

### Antibacterial activity of Ac-Var-1 against Escherichia coli and Staphylococcus aureus

An agar diffusion assay was conducted to evaluate the antibacterial activity of Ac-Var-1 against gram-negative *E. coli* and gram-positive *S. aureus*. The negative control represented by the elution buffer alone did not show any zone of inhibition and was excluded from the graph. For *E. coli*, the positive control, ampicillin, exhibited a zone of inhibition with a diameter of 27.8 mm. The fusion protein Trx-Ac-Var-1 and formic acid-treated fusion protein (Trx-Ac-Var-1 + formic acid) demonstrated zone of clearance values of 20.5 mm and 23.8 mm, respectively. The active peptide fraction active Ac-Var-1 exhibited zone of clearance values of 27.4 mm while the peptide controls Epi-1 and Var-1 exhibited 25.1 and 27.2 mm, respectively. In the case of *S. aureus*, ampicillin exhibited a zone of inhibition of 25.8 mm. Trx-Ac-Var-1 and Trx-Ac-Var-1 treated with formic acid showed zone of clearance values of 18.3 mm and 22.2 mm. The active peptide Ac-Var-1, Epi-1, and Var-1 exhibited zone of clearance values of 26.2 mm, 23.3 mm, and 26.1 mm.

**Fig. 6:**
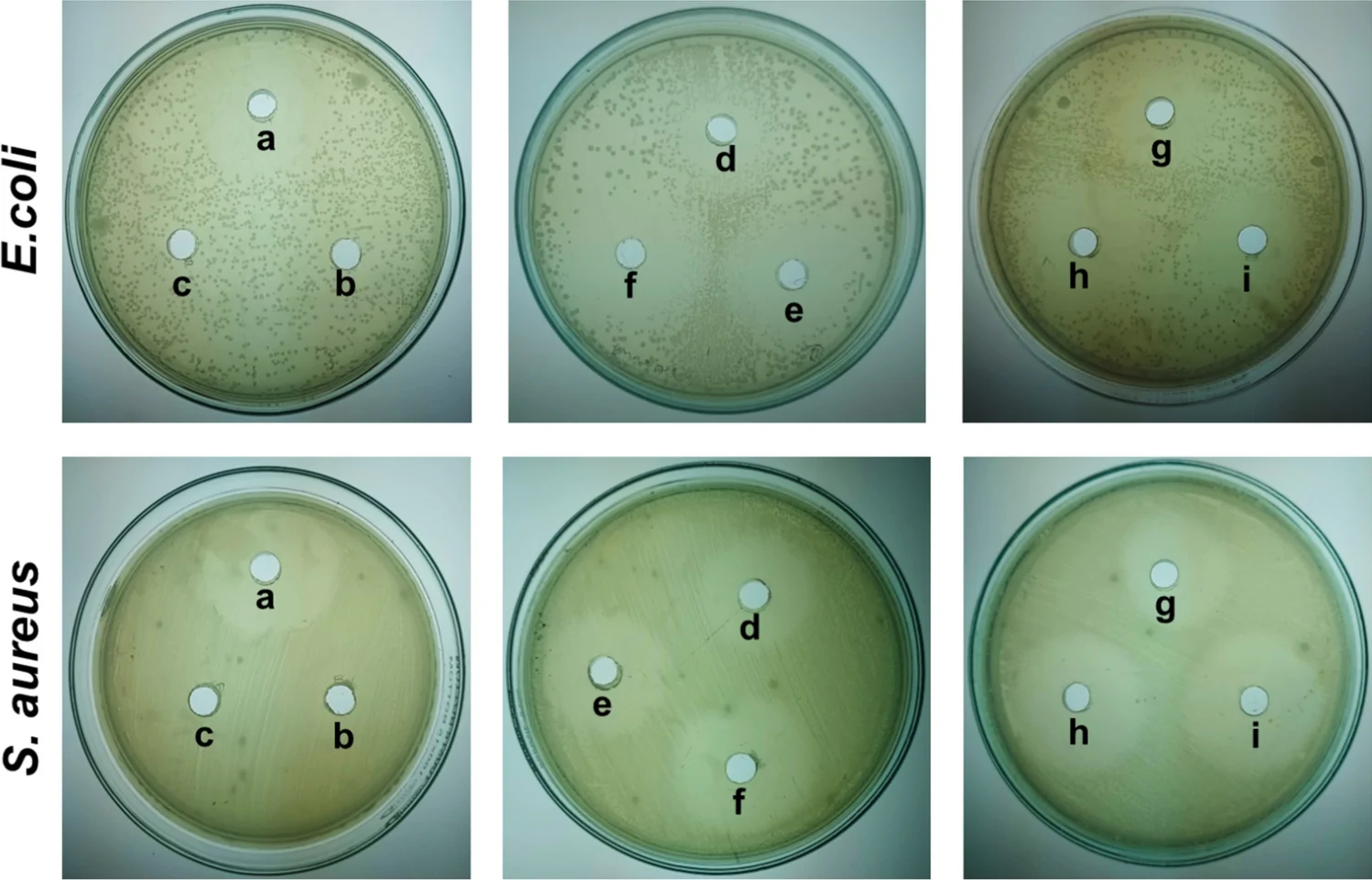
Agar well diffusion assay against pathogenic *E. coli* (MTCC 2939) and *S. aureus* (ATCC 25923). The wells are (**a**) ampicillin: positive control, (**b**) thioredoxin protein alone, (**c**) buffer: negative control, (**d**) Trx-Ac-Var-1, (**e**) Trx-Ac-Var-1 treated with formic acid and desalted, (**f**) Active Ac-Var-1, (**g**) Var-1, and (**h**) Active Ac-Var-1

**Fig. 7:**
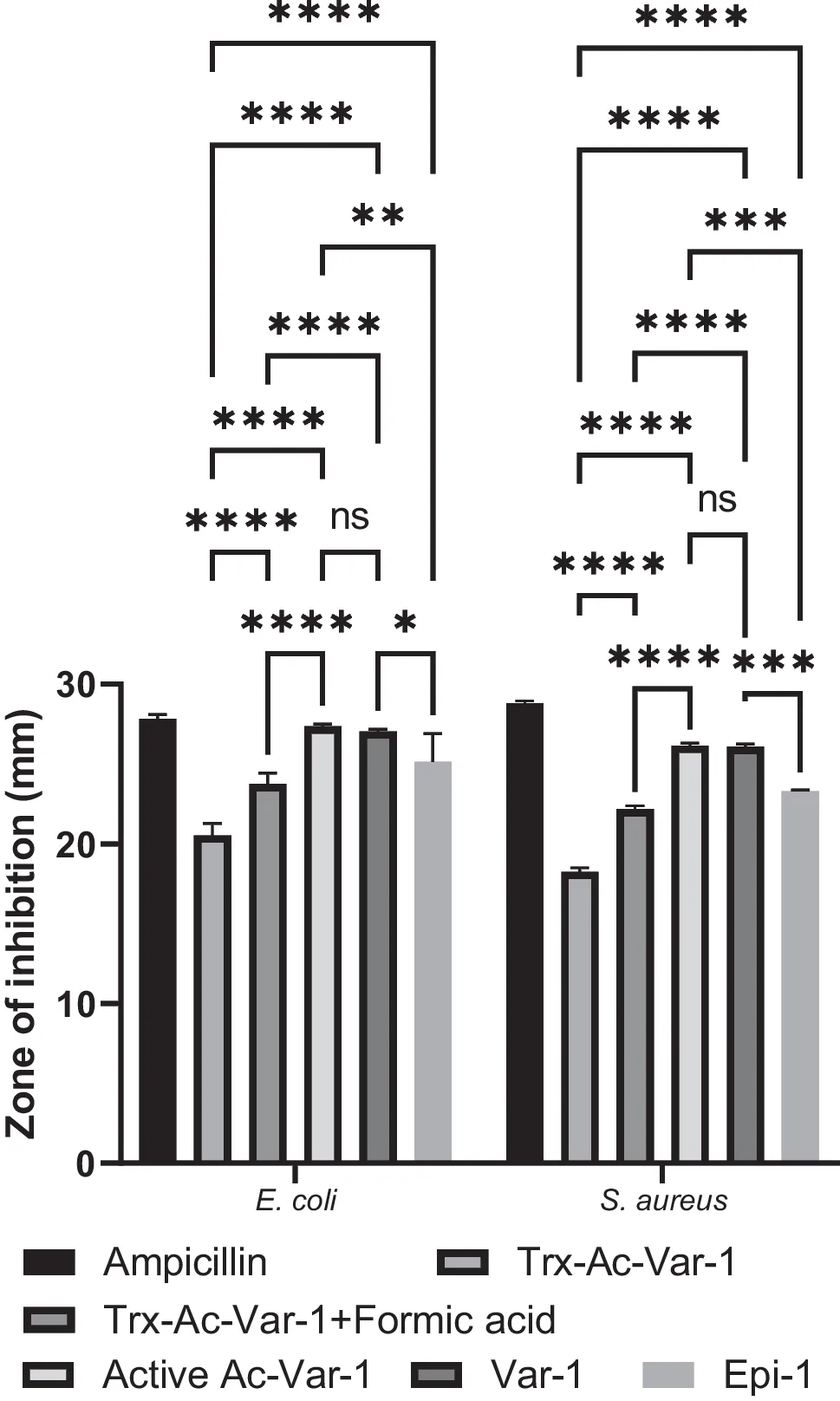
Graph showing the quantitated zone of inhibition for the photographed image. Each experiment was performed in triplicate, and the results are presented as the mean ± standard deviation. Tukey’s two-way ANOVA was used to compare the statistics between each parameter except for ampicillin. Statistical significance denoted are as follows: *p < 0.5, **p < 0.1, ***p < 0.01, and ****p < 0.001. ns denotes non-significant

An important finding in this study was that the acid-cleaved Trx-Ac-Var-1 demonstrated notably larger zones of inhibition compared to their corresponding thioredoxin fusion protein among the analyzed pathogens. In addition, the diameter of zone of inhibition for the active peptide Ac-Var-1 has more or less the same value like the Var-1 suggesting that the acid-cleaved Ac-Var-1 has the same efficiency like Var-1. Also, the Ac-Var-1 and Var-1 have bigger zone compared to wild-type epinecidin-1 (Figs. 6 and 7).

## Discussion

The demand of peptides in the pharmaceutical industry is in excess. Peptides are routinely produced by solid-phase chemistry for high-value fine chemical applications such as pharmaceuticals and functional therapeutic materials. Chemical synthesis is rapid and effective to produce custom-made peptides in relatively small quantities, but it is expensive and difficult during the scale-up process. Also, as the peptide sequence length increases to over 35 amino acids, it may not have a proper folded structure which is necessary for antimicrobial activity. Besides chemical synthesis of large peptides is expensive, the process also requires a large amount of organic solvent that presents potential environmental hazards. An alternative for expensive chemical synthesis is isolating the peptides from their natural source. However, a major drawback of isolating peptides from natural sources is kilograms of starting materials (such as tissues or secretions) are required to obtain a microgram quantity of the desired peptide. This protein quantity is insufficient to allow novel experiments or to use these peptides as a biological tool to treat infectious disease. Hence, recombinant production of peptides is ideally a better alternative compared to isolating peptides from natural sources and chemical synthesis. Recombinant systems are more effective to produce long peptides (> 35 amino acids) and proteins (Jeyarajan et al. 2010, 2023b; Govindarajan et al. 2012; Anbarasu and Sivakumar 2016). This technology uses a heterologous system called microbial “biofactories” (Ingham and Moore 2007). *Escherichia coli (E. coli)*, a Gram-negative bacterium, is an attractive system for producing heterologous proteins due to its rapid growth, low cost, and well-characterized genetics. The number of recombinant expressed antimicrobial peptides has greatly increased in recent years due to advances in molecular biology, which has allowed the establishment of strategies for host of choice, promoter type, and appropriate post-transcriptional modification. Once an optimal protocol for recombinant synthesis is established, it has the potential to provide large amounts of renewable and low-cost peptides. Nevertheless, achieving a high amount of recombinant peptides remains challenging.

It is challenging to express short polypeptides in *E. coli* due to the lytic activity of AMPs in the cytosol. To mask its lytic effects and enhance the expression of small peptides in the cytosol, a common approach is to co-express them with fusion partners such as thioredoxin (Jeyarajan et al. 2023a), ketosteroid isomerase (Panteleev and Ovchinnikova 2017), SUMO (Li 2009, 2011), and histone fold domain of the human transcription factor TAF12 domain (Vidovic et al. 2009). These proteins have a high-level expression in the bacterial host, so when small peptides are fused to these proteins, they function as carrier protein facilitating expression of the small peptides. Thioredoxin is known to be an extremely useful fusion partner protein, facilitating accurate disulfide-bond formation and expression of the desired recombinant protein. In our study, thioredoxin successfully entraps recombinant Ac-Var-1 and thereby prevents its intrinsic lytic activity against the host cell.

Along with the carrier proteins, purification tags such as GST (Glutathione S transferase), MBP (Maltose binding Protein), and 6 × His tag aid in affinity purification. Fusion co-expression requires specific enzymatic or chemical cleavage sites to release the active peptide. Enzymatic digestions using factor Xa (IEGR^↓^), enterokinase (DDDDK^↓^), thrombin (LVPR^↓^GS), TEV protease (ENLYFQ^↓^G), and HRV 3C protease (LEVLFQ^↓^GP) are commonly used methods for hydrolyzing fusion proteins (Partridge and Davis 1955). Although these methods are highly specific, they have limitations in terms of enzyme costs and sensitivity to environmental changes. Fusion protein purified under denaturing conditions can affect cleavage enzyme activity and peptides often tend to form aggregates.

Alternatively, chemical cleavage offers advantages such as lower reagent costs, wider temperature and pH range, compatibility with denaturing agents, and shorter cleavage recognition sites. Certain peptide bonds are less stable under acidic conditions. Partridge and Davis (Partridge and Davis 1955) discovered the acid lability of –D–X– bonds (where X = any amino acid). Among –D–X– bonds, –D–P– and –D–C– were found to be the most acid-sensitive. The mechanism of –D–P– bond acid hydrolysis involves the N atom of proline attacking the protonated sidechain carboxyl of aspartate, forming an unstable cation-imide intermediate that rapidly undergoes hydrolysis. This ultimately relieves the active peptide from the carrier protein, The advantage of acid cleavage is that the aggregated peptides trapped in inclusion bodies can be processed without the need to renature the protein (Fjell et al. 2008).

Discussing the AMP sequences, most natural AMPs have not entered the drug market for therapeutic applications due to high manufacturing costs, poor pharmacokinetic properties, and low efficacy. To overcome these challenges, computational design techniques, such as Hidden Markov Models and template-based methods, are required to optimize AMP sequences and develop more active and less toxic antimicrobial peptides (Fjell et al. 2012; Ranjith et al. 2022). It has also been proved that many computational designed AMPs exhibit expected pharmacokinetic property (Thankappan et al. 2013) with selective killing towards bacterial cells with several orders of magnitude at low concentration without affecting the viability of the mammalian cells. This template-based strategy also helps to screen the antimicrobial peptide sequences for temperature resistance, salt resistance, protease resistance, shelf life, hemolytic activity, and cytolytic activity (Ranjith et al. 2022). Efforts have been directed to discern the physico-chemical parameters such as charge, hydrophobicity, and conformation which governed the mode and selectivity of AMPs. This physico-chemical code will influence the AMP properties such as spectrum of action, kill kinetics, and membrane permeabilizing abilities (Thankappan et al. 2013). Better AMP can be designed by interplaying these hierarchical factors which influence the specific function of AMPs alleviating the unwanted effects. From the available reports (Thankappan et al. 2021; Jeyarajan et al. 2023a), it can be decoded that AMP potency can be boosted by interplaying the amino acid sequence. It is often correlated with the presence of specific amino acids at certain position.

Improved AMPs have come with substitution of certain amino acids due to their unique chemical properties which make the AMPs, for more effective function. Inspired by the studies showing the substitution of lysine in natural AMPs increases the positive charge that facilitates the interaction with the anionic components of the bacterial membrane, we have previously designed variants of AMP epinecidin-1 for improved performance. We have shown that lysine substitution has aided increased antimicrobial activity (Jeyarajan et al. 2023a). The helical structural features pertaining to outer membrane permeabilization and kill kinetics have also been enhanced, endowing broad-spectrum action.

In our previous study (Jeyarajan et al. 2023a), we isolated the Var-1 peptide from the fusion Trx-Var-1 through enterokinase digestion. However, the fusion Trx-Var-1 became localized in the inclusion bodies. Consequently, the protein was solubilized and purified under denaturing conditions with His-tag affinity chromatography. After purification, the denaturant was removed through buffer exchange to enable the enterokinase enzyme to act on the enterokinase site. This process required additional time, effort, preliminary sourcing, and protocol development. Hence, we sought an alternative method to separate the active peptide, and acid cleavage proved to be a useful approach. The acid can act under denaturing conditions to release the active peptide, providing a significant advantage. Therefore, we introduced an acid-labile recognition site between thioredoxin and Var-1, which we named Ac-Var-1. Upon expression in the *E. coli* system with IPTG induction, the protein from this clone also localized in the inclusion bodies. Subsequently, the fusion Trx-Ac-Var-1 was purified under denaturing conditions and cleaved directly using 50% formic acid without the need for renaturation or buffer exchange. Following acid cleavage, the active Ac-Var-1 peptide was purified and characterized using mass spectrometry. The analysis indicated that Ac-Var-1 was produced entirely intact, with its molecular weight precisely matching the calculated value. This observation also demonstrates the heat, salt, and acid tolerance of the peptide.

One thing to note is that although the active Ac-Var-1 is released, the carrier protein thioredoxin is observed as a contaminant in the SDS-PAGE. This may be attributed to the binding of Ac-Var-1 to thioredoxin and vice versa, as the peptide exhibits a high potential for protein binding. Additionally, the pI of Ac-Var-1 is 10.61, which could facilitate binding to negatively charged amino acids in the carrier protein. There are also reports indicating unsuccessful attempts to separate the carrier protein from the active peptide (McKinney et al. 2004; Gibbs et al. 2004; Zhou et al. 2007; Kim et al. 2007).

This study investigated antimicrobial activity against pathogens such as gram-negative *E. coli* and gram-positive *S. aureus*, both of which are known to cause 62% of bloodstream infections (BSI) (De and Angelis et al. 2018), particularly affecting patients in the Intensive Care Unit (ICU). *E. coli*, with a larger prevalence among BSI patients, results in unfavorable prognoses and increased hospital mortality (Zaidenstein et al. 2018). *S. aureus*, resistant to methicillin and vancomycin, leaves patients susceptible to infections. Infections from *S. aureus* can range from skin and soft tissue infections to severe conditions such as bacteremia, pneumonia, endocarditis, bone and joint infections, and even toxic shock syndrome (Peacock and Paterson 2015). The treatment of these deadly infections can be challenging, therefore, necessitating an effective alternative. Antimicrobial peptides (AMPs) have the potential to fulfill this requirement. Ac-Var-1 has been seen to impede the growth of both *E. coli* and *S. aureus*, signifying its potential usage as an adjunctive agent with antibiotics to combat infection. Furthermore, Ac-Var-1 showed enhanced activity when compared to the wild-type epinecidin-1.

Optimizing the separation of thioredoxin itself represents a significant area of study. Successfully achieving this separation could pave the way for the recombinant production of numerous antimicrobial peptides (AMPs). One potential approach is to leverage the strong positive charge of the peptide by strategically selecting negatively charged resins and optimizing solvent and salt conditions. This approach may yield a successful separation between the carrier protein and the active AMP, addressing the issue of the fusion protein being a contaminant in the active peptide preparation.

In conclusion, the present study presents an efficient strategy for the biotechnological production of the epinecidin-1 variant Ac-Var-1. This study emphasizes the advantages, challenges, and methodology involved in obtaining an active antimicrobial peptide in a heterologous system.

## Data Availability

The data sets generated or analyzed during this current study are included in this article.
